# Sub-toxic Ethanol Exposure Modulates Gene Expression and Enzyme Activity of Antioxidant Systems to Provide Neuroprotection in Hippocampal HT22 Cells

**DOI:** 10.3389/fphys.2016.00312

**Published:** 2016-07-27

**Authors:** Verónica Casañas-Sánchez, José A. Pérez, David Quinto-Alemany, Mario Díaz

**Affiliations:** ^1^Departamento de Bioquímica, Microbiología, Biología Celular y Genética, Universidad de La LagunaTenerife, Spain; ^2^Instituto Universitario de Enfermedades Tropicales y Salud Pública de CanariasTenerife, Spain; ^3^Departamento de Biología Animal, Edafología y Geología, Universidad de La LagunaTenerife, Spain; ^4^Unidad Asociada de Investigación ULL-CSIC, “Fisiología y Biofísica de la Membrana Celular en Patologías Neurodegenerativas y Tumorales”Tenerife, Spain

**Keywords:** ethanol, antioxidant systems, superoxide dismutases, glutathione, thioredoxins, hippocampal cells, HT22 cells

## Abstract

Ethanol is known to cause severe systemic damage often explained as secondary to oxidative stress. Brain is particularly vulnerable to ethanol-induced reactive oxygen species (ROS) because the high amounts of lipids, and because nerve cell membranes contain high amounts of peroxidable fatty acids. Usually these effects of ethanol are associated to high and/or chronic exposure to ethanol. However, as we show in this manuscript, a low and acute dose of ethanol trigger a completely different response in hippocampal cells. Thus, we have observed that 0.1% ethanol exposure to HT22 cells, a murine hippocampal-derived cell line, increases the transcriptional expression of different genes belonging to the classical, glutathione/glutaredoxin and thioredoxin/peroxiredoxin antioxidant systems, these including *Sod1, Sod2, Gpx1, Gclc*, and *Txnrd1*. Paralleling these changes, enzyme activities of total superoxide dismutase (tSOD), catalase, total glutathione peroxidase (tGPx), glutathione-*S*-reductase (G*S*R), and total thioredoxin reductase (tTXNRD), were all increased, while the generation of thiobarbituric acid reactive substances (TBARS), as indicators of lipid peroxidation, and glutathione levels remained unaltered. Ethanol exposure did not affect cell viability or cell growing as assessed by real-time cell culture monitoring, indicating that low ethanol doses are not deleterious for hippocampal cells, but rather prevented glutamate-induced excitotoxicity. In summary, we conclude that sub-toxic exposure to ethanol may well be neuroprotective against oxidative insults in hippocampal cells.

## Introduction

Ethanol is known to induce neurocognitive deficits and to provoke neuronal injuries associated with neuronal degeneration (Givens et al., 2000). Although oxidative stress and mitochondrial damage are implicated in nerve tissue injury (Suh et al., 2004; Das and Vasudevan, 2007) the precise mechanisms underlying ethanol-induced neurological disorders remain unclear. Ethanol-induced oxidative stress is linked to its metabolism in both microsomal and mitochondrial systems, which is directly involved in the generation of reactive oxygen species (ROS) and reactive nitrogen species (Das and Vasudevan, 2007). It is known that high or chronic ethanol exposure results in the depletion of reduced glutathione (GSH) levels, decreases antioxidant activity, and elevates malondialdehyde and hydroxynonenal protein adducts (Das and Vasudevan, 2007). These increased levels of oxidative stress (and secondary membrane lipid peroxidation, in particular) may disrupt neuronal energy metabolism and ion homeostasis by impairing the function of membrane ion-motive ATPases and glucose and glutamate transporters. Such oxidative and metabolic disturbances may thereby render neurons vulnerable to excitotoxicity and apoptosis. Indeed, it is known that acute alcohol exposure inhibits cognitive functions, including learning, and memory in humans (Givens et al., 2000), and that ethanol-induced memory impairments are mainly due to deficits in processing new memories, rather than retrieval of consolidated memories (White et al., 2000). The hippocampus is the main locus for ethanol-induced alterations of cognitive functions. Compelling evidence have demonstrated that acute ethanol exposure inhibits long-term potentiation (LTP) in the CA1 region of the hippocampus both *in vivo* and *in vitro* (Givens and McMahon, 1995; White et al., 2000; Ramachandran et al., 2015). Apparently, the primary mechanism by which ethanol inhibits LTP is the increase in GABAergic transmission, which, in turn, inhibits the depolarization phase required for N-methyl-D-aspartate (NMDA) receptor activation (White et al., 2000; Schummers and Browning, 2001).

Apart from these deleterious effects, several lines of evidence have shown that consumption of low doses of alcohol may provide neuroprotective effects against Alzheimers disease (AD). Indeed, Belmadani et al. (2004) observed that acute administration of ethanol was able to prevent the toxic effects of amyloid beta peptides (Aβ), the main protein aggregates in Alzheimers disease, in brain slices. Pre-treatment with physiologically relevant concentrations of ethanol (0.02–0.08%) protected neurons against Aβ-induced synapse damage, and recovered levels of synaptophysin, an indicator of synapse density in cortical and hippocampal neurons (Bate and Williams, 2011). Further, it has been reported that moderate wine consumption reduced neuropathologic traits (decreased amyloid plaques and reduced spatial memory impairment), in Tg2576 transgenic mice which overexpress amyloid pre-cursor protein (Wang et al., 2006). Moreover, epidemiological studies in a large cohort of subjects (>3600) have pointed out that low or moderate alcohol consumption is associated with lower risk of incident dementia among older adults, and these individuals are less likely to develop phenotypic symptoms of Alzheimers disease (Mukamal et al., 2003). Interestingly, ethanol also protected neurons against synapse damage induced by pre-synaptic aggregates of α-synuclein, which are characteristic of Parkinson's disease and dementia with Lewy bodies (Bate and Williams, 2011).

The potential neuroprotective mechanism(s) of action of ethanol remain unknown. However, given that amyloid beta peptides and α-synuclein toxicities have been linked to increased oxidative stress (Simonian and Coyle, 1996; Mattson et al., 1999; Bossy-Wetzel et al., 2004; Mancuso et al., 2006), in the present study we have examined a possible mechanism by which moderate ethanol concentrations might exert a neuroprotective effect on hippocampal cells through modification of cellular antioxidant capabilities. We demonstrate for the first time, that sub-toxic ethanol exposure triggers the activation of cellular antioxidant systems and that its effects may be unraveled both at transcriptional and enzymatic levels. Our results demonstrate that, at low doses, ethanol exerts a role as an “*Indirect Antioxidant*,” at least in hippocampal cells.

## Materials and methods

### Cell culture conditions, ethanol supplementation and preparation of cell extracts

The immortalized mouse hippocampal cell line, HT22, was cultured in standard Dulbecco's modified Eagle's medium (DMEM) as described in Martín et al. (2006). Culture medium was changed every 2 days and, when reached 90% confluence, cells were subcultured after treatment with 0.25% trypsin-EDTA mixture, at a density of 4 × 10^5^/ml on T25 flasks (for gene expression analyses) and T75 flasks (for enzyme activity studies).

After allowing 24 h for attachment, culture media were replaced with standard medium supplemented with ethanol in a 0.1% final concentration or vehicle phosphate buffered saline (PBS). Every 24 h of incubation the medium was replaced with fresh medium containing ethanol in PBS or PBS. Cell cultures were collected at 6, 24, 30, and 48 h, and immediately processed for either total RNA extraction or preparation of total extracts required for determination of enzyme activities, and glutathione and TBARs levels.

Cell extracts were prepared by homogenization in cold hypotonic buffer (Tris-HCl 20 mM, pH = 7.6) containing 1X protease inhibitors cocktail (Roche Diagnostics, Barcelona, Spain), then centrifuged at 900 g for 10 min, and the supernatants collected and stored at −80°C until analyses. For glutathione measurements, cell extracts were homogenized in cold hypotonic buffer containing 5% TCA and centrifuged at 10,000 g for 10 min at 4°C to isolate the post-mitochondrial supernatant. Supernatants were stored in 150 μl aliquots at −80°C. Protein determination was carried out using the Bradford assay (Bradford, 1976).

### RNA purification, cDNA synthesis and relative quantification of gene expression

Total RNA was purified from 3 × 10^6^ HT22 cells using a commercial kit and following the manufacturer's recommendations (RNeasy®Protect Minikit, Qiagen), and on-colum DNase I digestion to remove genomic DNA (gDNA). The integrity of purified RNA was estimated through the 3′:5′ assay (Nolan et al., 2006).

cDNA samples for real-time reverse transcription quantitative PCR (RT-qPCR) experiments were obtained with the Transcriptor First Strand cDNA Synthesis Kit (Roche), using 6 μg total RNA as template and anchored oligo(dT)^18^ primers. A mixture of the 33 diluted cDNA samples was used for the selection of the optimal concentration of each PCR primer pair (see Table 1), based on the lowest quantification cycle (Cq) values. Resulting amplicons for each primer pair from the cDNA pool were checked by electrophoresis on 3% agarose gels and sequenced. The possibility of gDNA contamination in the RT-qPCR assays was controlled in several ways. First, amplification primers were targeted to different exons, often spanning an exon/exon boundary (see Table 1). Next, each primer pair was tested by real-time qPCR using 1 ng genomic DNA as template. The level of gDNA contamination in each of the 32 RNA samples was assessed, assaying a quantity equivalent to the cDNA used in the amplification reactions (i.e., 40 ng of total RNA) was amplified by real-time qPCR using primers targeted to alpha-tubulin sequences.

**Table 1:** **Gene names, cellular locations, and oligonucleotides used as primers in amplification reactions**.

**Gene**		**mRNA**	Comments^*^cellular location	**Amplification primers**		**Amplicon size (bp)**	
**Product**	**Symbol**	**Reference sequence**		**Forward/Reverse**	**Targeted exons**	**cDNA**	gDNA^**^
Hypoxanthine guanine phosphoribosyl transferase	*Hprt*	NM_013556	IS	TCAGACTGAAGAGCTACTGTAATGA/AAGTTTGCATTGTTTTACCAGTG	3rd/4th 6th	136	N/A
TATA binding protein	*Tbp*	NM_013684	IS RIA (3′')	GACCCACCAGCAGTTCAGTAG/CTCTGCTCTAACTTTAGCACCTGT	6th 7th/8th	136	N/A
TATA binding protein	*Tbp*	NM_013684	RIA (5′')	CGCAGTGCCCAGCATCA/GCATAAGGTGGAAGGCTGTTG	1st/2nd 2nd	154	N/A
α-Tubulin	Tuba1	NM_011653	gDNA contamination control	GGATTCGCAAGCTGGCTG/GGGCTGGGTAAATGGAGAAC	3rd/4th 4 h	162	162
RNA Polimerase II (subunit f)	Polr2f	NM_027231	IS	GTCAGACAACGAGGACAATTTC/ATACTTGGTCATGTAAGGAGTGGT	1st/2nd 3rd	178	N/A
Superoxide dismutase 1	*Sod1*	NM_011434	Cytosolic	CGATGAAAGCGGTGTGCG/GCACTGGTACAGCCTTGTGTATTG	1st2nd/3rd	178	N/A
Superoxide dismutase 2	*Sod2*	NM_013671	Mitochondrial	GGTGGAGAACCCAAAGGAGA/TAAGCGACCTTGCTCCTTATT	3rd/4th 4 h	150	N/A
Catalase	*Cat*	NM_009804	Peroxisomal	AGAGGAAACGCCTGTGTGAG/GTAGGTGTGAATTGCGTTCTTAG	11th12th/13th	173	N/A
Thioredoxin 1	*Txn1*	NM_011660	Cytosolic	AGCCCTTCTTCCATTCCCT/GAACTCCCCCACCTTTTGAC	2nd73rd 4th/5th	152	152
Thioredoxin 2	*Txn2*	NM_019913	Mitochondrial	GACACCAGTTGTTGTGGACTTTC/TAGGCACAGCTGACACCTCATA	1st 2nd/3rd	170	N/A
Thioredoxin interacting protein	*Txnip*	NM_023719	Cytosolic	GTCGAATACTCCTTGCTGATCTA/TCTGGGGTATCTGGGATGTT	5th/6th 6th	173	N/A
Thioredoxin reductase 1	*Txnrd1*	NM_001042523	Cytosolic	GGGGAAGAAAATATTGAAGTTTACC/TGGAAGCCCACGACACGTT	13th/14th 15th	134	N/A
Thioredoxin reductase 2	*Txnrd2*	NM_013711	Mitochondrial	TCACAGTGCTACATAAAGATGGTA/CAGCTTGACCACCTCCTCAG	15th/16th 17th	192	N/A
Thioredoxin reductase 3	*Txnrd3*	NM_153162	Endoplasmic reticulum	GACTCTTTGGGGTCTCTTTAGAA/CAAAACAAGGTGTGATACACTTCC	12th 13th/14th	154	N/A
Peroxiredoxin 2	*Prdx2*	NM_011563	Cytosolic	GGCATTGCTTACAGGGGTC/CCACATTGGGCTTGATGGT	4th/5th 6th	196	196
Peroxiredoxin 3	*Prdx3*	NM_007452	Mitochondrial	GGATCAACACACCAAGAAAGAAT/CGGAAGGTCGTTGACACTCAG	4th/5th 6th	185	N/A
Peroxiredoxin 4	*Prdx4*	NM_016764	Cytosolic/Nuclear Secreted	GTATACCTTGAAGACTCAGGACATAC/CCAGCAGGGCAGACTTCTC	4th5th/6th	176	N/A
Peroxiredoxin 5	*Prdx5*	NM_012021	Cytosolic/Mitochondrial/peroxisomal	CACCTGGCTGTTCTAAGACCC/AGACACCAAAGAATCATCCAATAA	2nd/3rd 5th	224	N/A
Glutamate-cysteine ligase, catalytic subunit	*Gclc*	NM_010295	Cytosolic	GCCTCCTCCTCCAAACTCAGA/ATCCCCTGCAAGACAGCATC	11th 13th	213	N/A
Glutathione reductase	*Gsr*	NM_010344	Cytosolic/Mitochondrial	CCAATGTCAAAGGCGTCTATG/AGACCACAGTAGGGATGTTGTCA	10th 11th	153	N/A
Glutaredoxin 1	*Glrx1*	NM_053108	Cytosolic	AGCTCACCGGAGCGAGAAC/ATCTGCTTCAGCCGAGTCATC	1st 2nd	118	N/A
Glutaredoxin 2	*Glrx2*	NM_001038592	Cytosolic/Mitochondrial	GTCGTTTTGGGGGAAGTCTA/GGTTGCCATATTCCAGCATAT	2nd 3rd	182	N/A
Glutathione peroxidase 1	*Gpx1*	NM_008160	Cytosolic	CGGCACAGTCCACCGTGTAT/ATTCTTGCCATTCTCCTGGTGT	1st 1st/2nd	236	236
Glutathione peroxidase 4	*Gpx4*	NM_008162	Cytosolic/Mitochondrial	TGGTCTGGCAGGCACCAT/TGCACACGAAACCCCTGTACT	1st 3rd/4th	128	128
Sulfiredoxin 1	*Srxn1*	NM_029688	Cytosolic	AGAGCCTGGTGGACACGAT/AGCTTGGCAGGAATGGTCT	1st 2nd	163	N/A

* IS, internal standard for normalization; RIA, RNA integrity assessment by 3 ′:5 ′ assay. Subcellular localization of protein encoded by the detected mRNA variant.

** N/A: the primer pair does not amplify with gDNA as template, at least with a short extension time amplification reactions.

Real-time amplifications were run in triplicate using SYBR Green detection on a LightCycler 480 platform (Roche). Relative quantities of the targeted mRNAs were calculated from Cq data following an efficiency-correction model implemented in the REST software (Pfaffl, 2001). The normalization factor for each cDNA sample was calculated as the geometric mean of the expression values of reference genes *Hprt1, Polr2f*, and *Tbp* genes.

### Antioxidant enzyme activities and levels of glutathione and thiobarbituric acid reacting substances (TBARS)

Superoxide dismutase (SOD) activity was measured using the pyrogallol method following Marklund and Marklund (1974). One unit was defined as 50% inhibition of the rate of autoxidation of pyrogallol. The activity of SOD is expressed as units/mg protein. Catalase (CAT) activity was determined as described previously (Sani et al., 2006), by following the rate of decomposition of H_2_O_2_ in 10 mM potassium phosphate buffer at 240 nm. One CAT unit was defined as the decomposition of 1 mmol H_2_O_2_/min, and was expressed as units/mg protein.

Total glutathione peroxidase (GPX) and phospholipid-hydroperoxide glutathione peroxidase (GPX4) activities were measured using the glutathione reductase-NADPH methods described by Lawrence and Burk (1976) and Scheerer et al. (2007), respectively, by monitoring the rate of decrease in the concentration of NADPH as recorded at 340 nm. Glutathione reductase (G*S*R) was analyzed by determining the reduction of oxidized glutathione (GSSG) at the expenses of NADPH oxidation and monitored at 340 nm, according to the method of Carlberg and Mannervik (1985). GPX, GPX4 and GSR activities were expressed as nmol NADPH oxidized/min.mg protein.

Glutathione-*S*-transferase (G*S*T) activity was determined following the conjugation of GSH with CDNB (1-chloro-2,4-dinitrobenzene) at 340 nm (Habig et al., 1974) and expressed as nmol GS-DNB/min.mg protein.

Thioredoxin reductase (TXNR) activity was measured using the DTNB-NADPH assay described by Arnér et al. (1999), in which the generation of thionitrobenzoate ion (TNB) catalyzed by TXNR upon oxidation of NADPH is monitored at 412 nm. TXNR activity was expressed as nmol TNB/min.mg protein.

Glutathione levels were determined fluorimetrically using excitation/emission wavelengths of 355 nm/420 nm according to Hissin and Hilf (1976). GSH, GSSG, and total glutathione (GSH+2GSSG) levels were determined against proper calibration curves and expressed as nmol/mg protein. Lipid peroxidation was determined by the thiobarbituric acid reacting substances (TBARs) method (Ohkawa et al., 1979), using TMP (1,1,3,3-tetramethoxypropane) as standard for calibration curves. TBARS were measured fluorimetrically with 485 nm (excitation)/ 535 nm (emission) wavelengths. TBARS contents were expressed as nmol/mg protein.

### Real-time cell proliferation assays

Real-time cell proliferation studies were performed using the xCELLigence biosensor technology (Roche), an electrical impedance-based system that allows for the measurement of real-time cell proliferation (Ke et al., 2011). Briefly, HT22 cells were trypsinized, and seeded at a density of 3.5 × 10^3^ cells/well into 3 independent E16—xCELLigence plates. After an initial stabilization period, the impedance was recorded at 15 min intervals along the experiment, and the values converted to Cell Index (CI), a measure of the degree of cellular adhesion to the multi-electrode array. Generally, cell number directly correlates with output CI reading until confluency is achieved (CI_max_). 48 h after seeding, 10 μl of vehicle (PBS), or ethanol (final concentration 0.1%) were added to each well, and incubated for additional 24 h before replacing the incubating solutions with either DMEM, DMEM+0.1% EtOH, or DMEM+1% EtOH (see **Figure 5A**). In some experiments, glutamate (20 mM final concentration) was added during the last medium replacement. The concentration of glutamate chosen for these experiments was calculated as the IC50 obtained in the dose-response analyses performed using this same device (**Figure 5B**). For this purpose 24 h after initial attachment, cells were exposed to single doses of glutamate, ranging from 3 to 30 mM, while CI was continuously monitored. 24 h after exposure CI_max_ was obtained and used for logistic analyses.

### Statistics

Gene expression data were processed following an efficiency-corrected model for relative quantification (Pfaffl, 2001) and normalization with multiple internal controls as implemented in the qBASE software (Hellemans et al., 2007). Four genes showing high expression stability in relative expression analysis were tested as potential reference genes according to qBASE normalization tools, three of which were finally selected. A relative expression software tool (REST 2008, Pfaffl et al., 2002) was used to obtain the corresponding significance levels for each individual change in gene expression. Comparisons of gene expression levels between PBS and ethanol and expressed as fold values. *P*-values below 0.05 were considered statistically different.

Dose-response curves for glutamate were obtained from real-time cell proliferation assays. Data were fitted to a four-parameter logistic equation to obtain the IC50 value, using the software implemented in XCELLigence device.

Data from enzyme activity assays and glutathione/TBARs levels are expressed as mean ± SEM, and were analyzed by one-way ANOVA followed by Tukey's multiple comparison test. Comparisons between PBS and ethanol treatments at each time were performed using Student's *t*-test or Mann-Whitney *U*-test where appropriate.

## Results

### Effects of sub-toxic exposure to ethanol on the transcriptional activity of genes encoding for antioxidant systems

First, we analyzed the time-course of the effects of sub-toxic ethanol exposure (0.1%) on the expression of different genes encoding for antioxidant enzymes with relevant activity in neuronal cells. We explored key enzymes from the three major antioxidant systems, namely classical, thioredoxin/peroxyredoxin, and glutathione/glutaredoxin systems. Target genes analyzed are detailed in Table 1, together with the information on the primers used for PCR amplification.

Results in Table 2 show that superoxide dismutase encoding genes were up-regulated yet with different time-courses. Thus, while *Sod1* expression was stimulated just 6 h after each exposure to ethanol (0 and 24 h), *Sod2* was up-regulated after 30 h exposure to ethanol and remained so until the end of the experiment. The other gene relevant in the classical system, *Cat*, encoding for catalase, remained unaltered, or was down-regulated (at 24 h). Within the thioredoxin-peroxiredoxin system, *Txnrd1* gene, encoding for cytosolic thioredoxin reductase was soon up-regulated and its expression kept stimulated throughout the experiment. Paradoxically, *Txnrd2*, and *Txnrd3* genes, encoding for thioredoxin reductases from mitochondrial and endoplasmic reticulum isoforms, respectively, were down-regulated throughout the experiment, although only significantly for *Txnrd3*. A similar set of changes were observed for genes encoding for peroxyredoxins 2–5, all of which were significantly down-regulated yet with different time patterns. Finally, *Txnip* gene, encoding for the inhibitory thioredoxin interacting protein, was highly down-regulated, particularly 24 h after each addition of ethanol. No changes in the expression patterns were observed for either thioredoxin-encoding genes.

**Table 2:** **Gene expression of antioxidant systems in HT22 cells exposed to ethanol (0.1%) or PBS**.

**Ethanol vs. PBS**								
	**Ethanol 6 h**		**Ethanol 24 h**		**Ethanol 30 h**		**Ethanol 48 h**	
**Gene**	**Expression**	***p***	**Expression**	***p***	**Expression**	***p***	**Expression**	***p***
**CLASSICAL SYSTEM**								
*Sod1*	1.23^*^	**0.028**	1.09	0.300	1.26^*^	**0.009**	1.19	0.054
*Sod2*	1.15	0.275	1.18	0.070	1.41^*^	**0.004**	1.46^*^	**0.026**
*Cat*	0.98	0.722	0.74^*^	**0.014**	0.94	0.255	0.88	0.134
**THIOREDOXIN/PEROXIREDOXIN SYSTEM**								
*Txn1*	1.09	0.486	1.01	0.990	1.26	0.119	1.08	0.469
*Txn2*	0.80	0.180	1.08	0.404	1.02	0.935	0.96	0.560
*Txnip*	1.02	0.892	0.47^*^	**0.008**	0.86	0.137	0.30^*^	**0.017**
*Txnrd1*	1.16^*^	**0.050**	1.27^*^	**0.010**	1.53^*^	**0.005**	1.32^*^	**0.018**
*Txnrd2*	0.74^*^	**0.032**	0.85	0.250	0.84	0.161	0.76	0.094
*Txnrd3*	0.71^*^	**0.027**	0.70^*^	**0.044**	0.68^*^	**0.021**	0.65^*^	**0.004**
*Prdx2*	0.75^*^	**0.046**	0.71^*^	**0.015**	0.82^*^	**0.015**	0.73^*^	**0.015**
*Prdx3*	0.76	0.078	0.68^*^	**0.036**	0.84	0.310	0.73^*^	**0.029**
*Prdx4*	0.75^*^	**0.012**	0.67^*^	**0.039**	0.78^*^	**0.008**	0.67^*^	**0.013**
*Prdx5*	0.73^*^	**0.046**	0.69^*^	**0.007**	0.72^*^	**0.011**	0.61^*^	**0.019**
*Srxn1*	1.00	0.949	1.26	0.163	1.15	0.439	1.13	0.052
**GLUTATHIONE/GLUTAREDOXIN SYSTEM**								
*Gclc*	1.13	0.135	1.07	0.700	1.26^*^	**0.013**	1.25^*^	**0.008**
*Gsr*	1.00	0.971	1.02	0.694	1.14^*^	**0.007**	1.09	0.136
*Glrx1*	1.25^*^	**0.017**	1.01	0.795	1.21	0.208	0.93	0.619
*Glrx2*	0.94	0.475	0.87	0.408	1.03	0.687	0.87	0.069
*Gpx1*	1.70^*^	**0.011**	1.84^*^	**0.015**	2.04^*^	**0.018**	2.20^*^	**0.025**
*Gpx4*	0.96	0.799	0.97	0.699	0.94	0.864	0.85	0.394

Results correspond to the mean of four different experiments. PBS, phosphate buffer saline; p, probability value;

* Significant differences.

Bold values indicate those genes whose expression was altered at some stage in the experiments.

Lastly, within the glutathione/glutaredoxin system, all affected genes were up-regulated. The most important changes were observed for *Gpx1*, encoding for cytosolic glutathione peroxidase 1, which remained stimulated throughout the experiment, and reached the maximal transcriptional stimulation amongst all genes studied at 48 h. These changes were specific for *Gpx1*, and were not observed for *Gpx4* gene which encodes the membrane-associated isoform. Also, significant changes were detected for *Gclc* gene, which encodes for the catalytic subunit of glutathione-cysteine ligase.

### Effects of sub-toxic exposure to ethanol on key antioxidant enzyme activities and antioxidant metabolites

**Figure 1:**
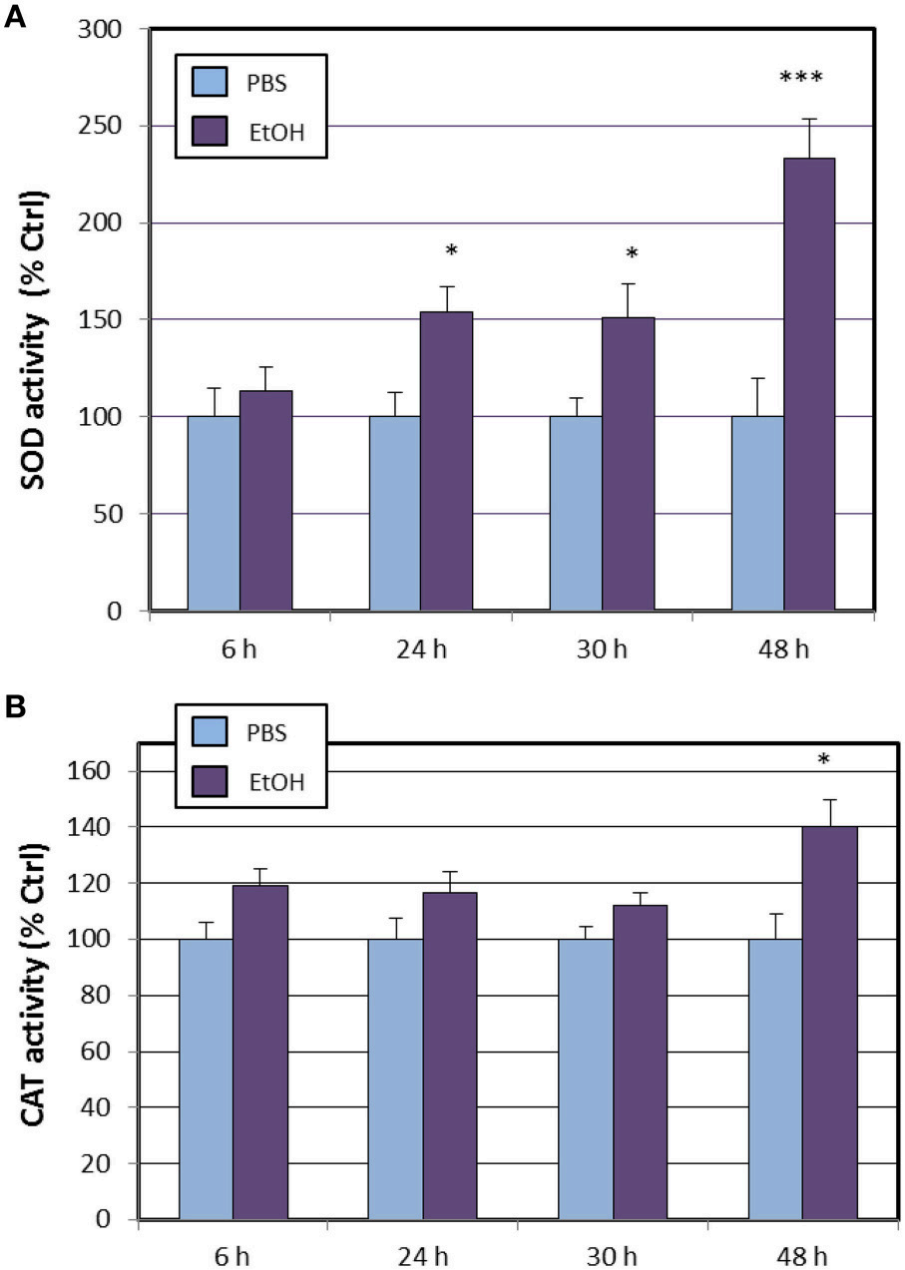
**Effects of ethanol (EtOH, 0.1%) on total SOD (A) and catalase (B) activities in HT22 cells at different times after first exposure to ethanol**. Results correspond to the mean ± SEM of four different experiments. ^*^, and ^***^p < 0.05 and *p* < 0.005 compared to vehicle (PBS), respectively.

Next, we assessed the activities of most antioxidant enzymes encoded by genes that were affected by ethanol treatment and following the same time-course used to explore changes in transcriptional activity. Results shown in Figure 1A shows that total superoxide dismutase activity was increased by ethanol treatment from 24 h after exposure, being the maximal activity reached at 48 h. Surprisingly, catalase activity remained similar to vehicle (PBS) until the end of the experiment, where at 48 h a significant increase (40%) was observed (Figure 1B).

**Figure 2:**
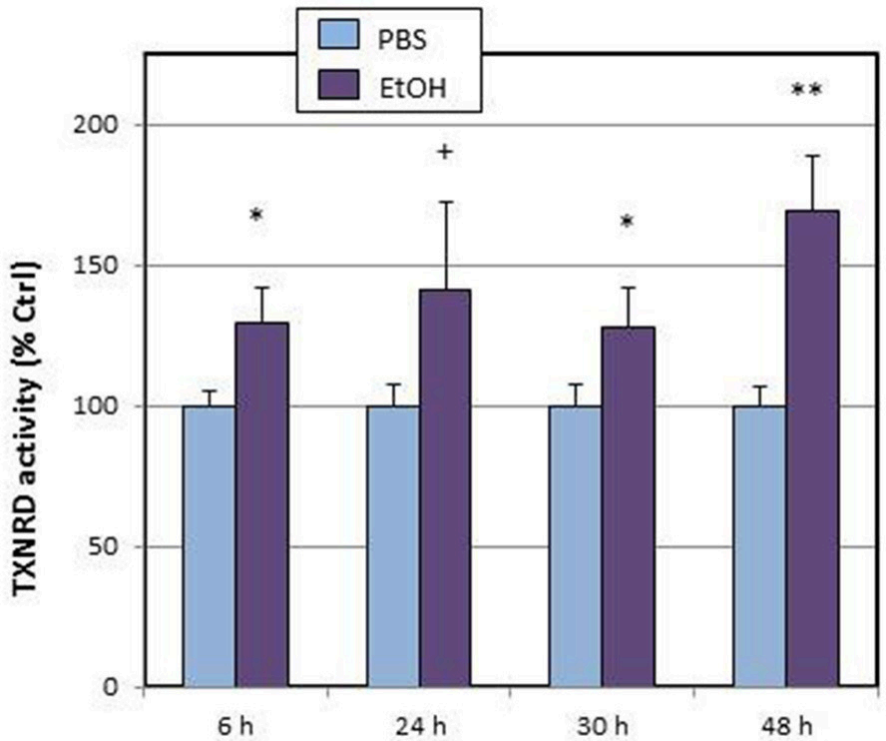
**Effects of ethanol (EtOH, 0.1%) on total TXNRD activity in HT22 cells at different times after first exposure to ethanol**. Results correspond to the mean ± SEM of four different experiments. ^+^, ^*^, ^**^p < 0.1, *p* < 0.05, and *p* < 0.01 compared to vehicle (PBS), respectively.

Within the thioredoxin system, we explored the time-course of total thioredoxin reductase (tTXNRD) activity (Figure 2). We observed that, in line with the results of *Txnrd1* up-regulation, TXNRD activity was stimulated from 6 h, and that maximal activation was observed at 48 h.

**Figure 3:**
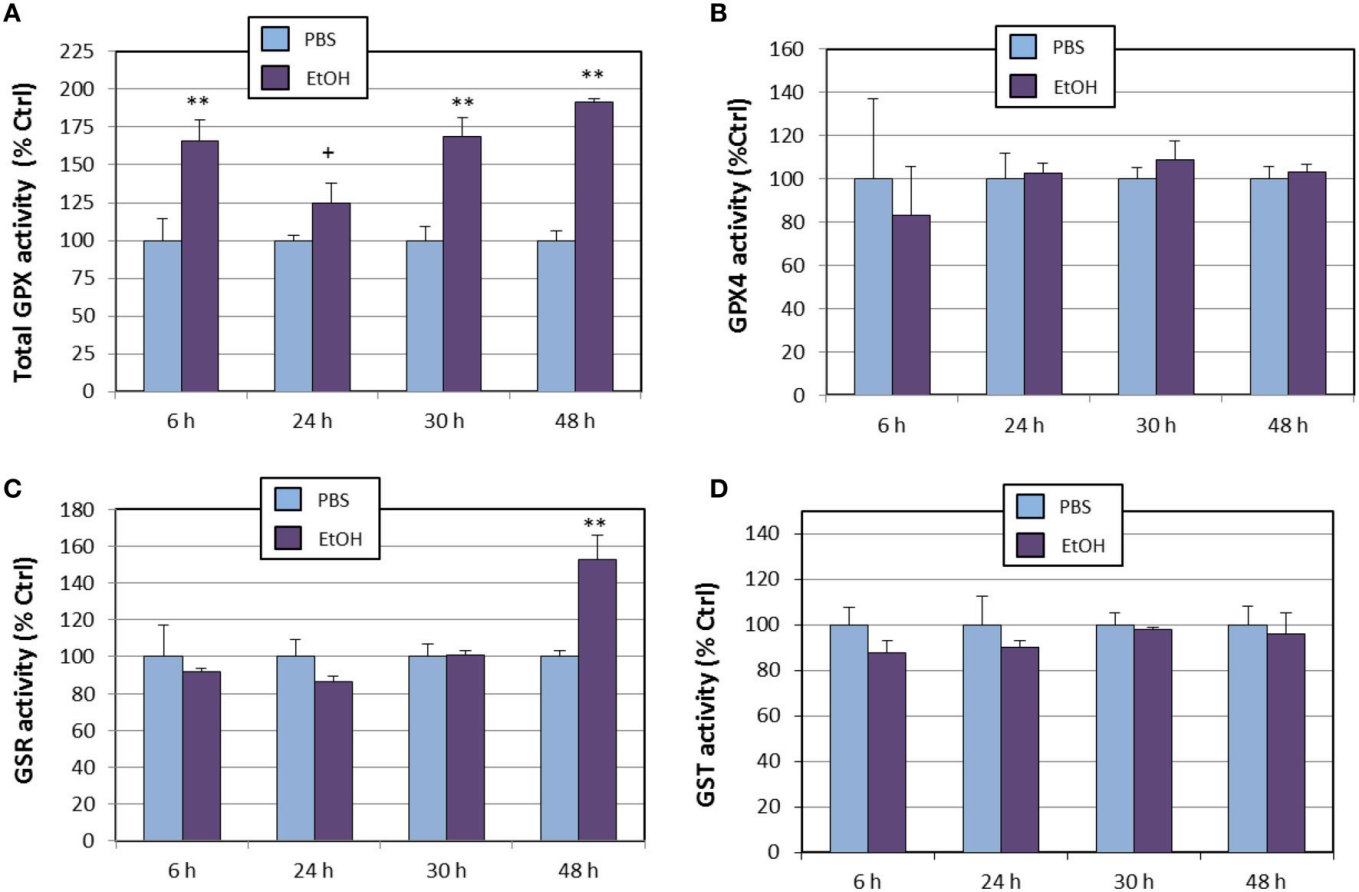
**Effects of ethanol (EtOH, 0.1%) on total GPX (A), GPX4 (B), G***S***R (C), and G***S***T (D) activities in HT22 cells at different times after first exposure to ethanol**. Results correspond to the mean ± SEM of four different experiments. ^+^, ^**^p < 0.1 and *p* < 0.01 compared to vehicle (PBS), respectively.

With regards to the glutathione/glutaredoxin system, we assessed the enzyme activities of total glutathione peroxidase, glutathione peroxidase 4, glutathione-*S*-reductase, and glutathione-*S*-transferase (Figure 3). When compared to PBS, total glutathione peroxidase (tGPx) was found to be significantly increased from the begin of the experiment (Figure 3A), reaching nearly a 200% increase at 48 h (similar to what was observed for *Gpx1* gene expression). This increase is likely attributable to stimulation of the GPX1 isoform, since GPX4 activity remained unaltered all along the experiment (Figure 3B). Glutahione-*S*-reductase (Figure 3C) was only affected at 48 h, which is compatible with the significant increase in *Gsr* gene expression detected at 30 h, and agrees with the expected delay in *Gsr* mRNA translation. On the other hand, glutathione-*S*-transferase was completely unaffected by ethanol treatment (Figure 3D).

**Figure 4:**
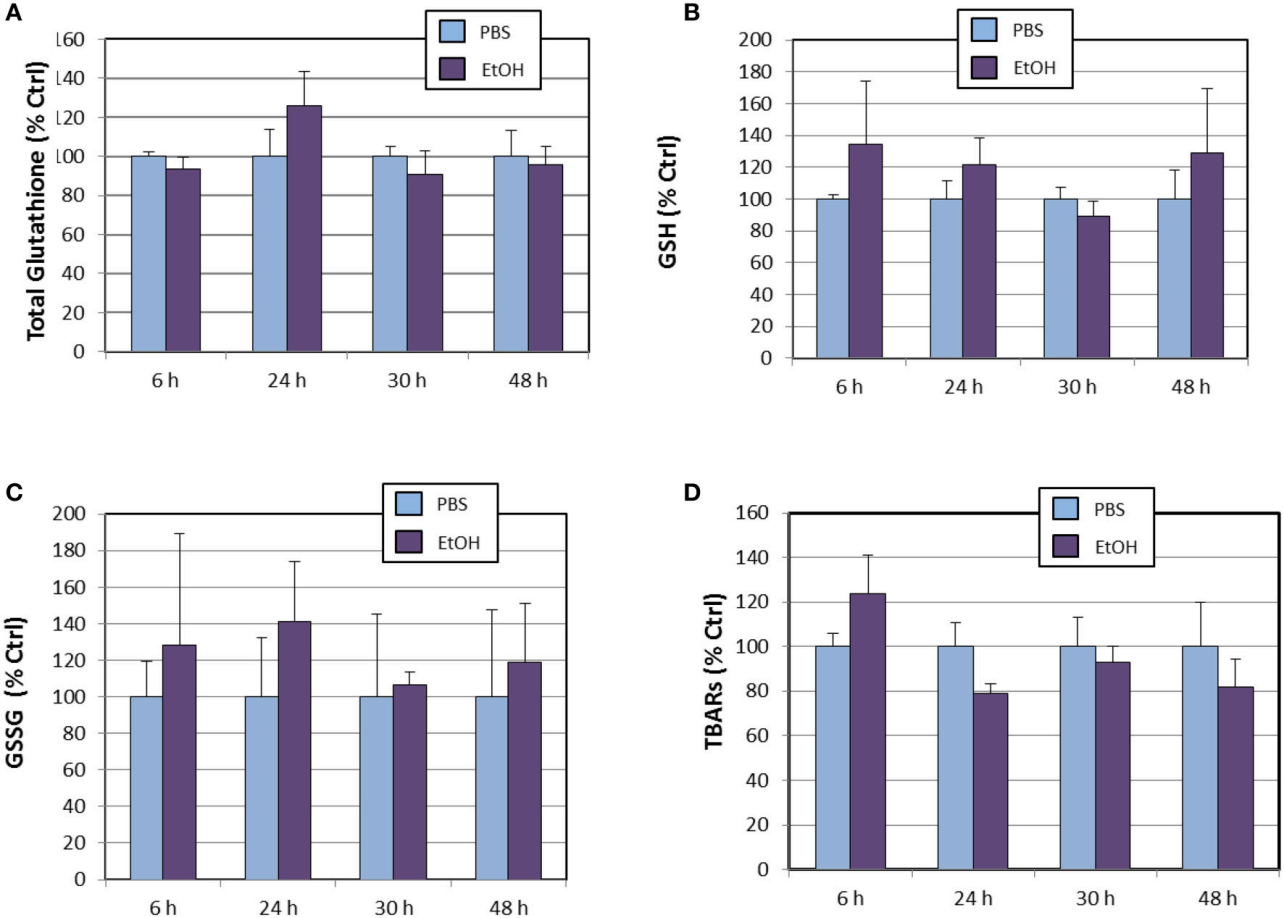
**Effects of ethanol (EtOH, 0.1%) on total glutathione (A), reduced glutathione (B), oxidized glutathione (C), and TBARs (D) levels in HT22 cells at different times after first exposure to ethanol**. Results correspond to the mean ± SEM of four different experiments.

We also determined cellular levels of total glutathione, reduced glutathione, oxidized glutathione (Figure 4). None of these oligopeptides appeared to be affected by ethanol treatment throughout the experiment, when compared to PBS-treated cells. These observations on glutathione species are in contrast to the expression levels of *Gclc* gene, which were significantly increased by the end of the experiment (Table 2). Several possible explanations are that even at 48 h, (1) *Gclc* mRNAs has not been fully translated, (2) that newly-synthetized GCLC protein might not be physiologically active or, (3) that expression of the regulatory subunit (GCLM) might be limiting, as demonstrated for HepG2/C3A cells and murine embryonic fibroblasts cultured in cysteine-deficient medium (Sikalidis et al., 2014). Regarding TBARs, it is noticeable that their levels did not increase at any point of the experiment, indicating that potential lipid peroxidation induced by ethanol is buffered by concerted activation of antioxidant systems. Indeed, although not significantly, there appear to occur a time-dependent reduction of TBARs levels in ethanol-treated cells (Figure 4D).

### Effects of ethanol on the time-course of HT22 cell proliferation and resistance to excitotoxicity

**Figure 5:**
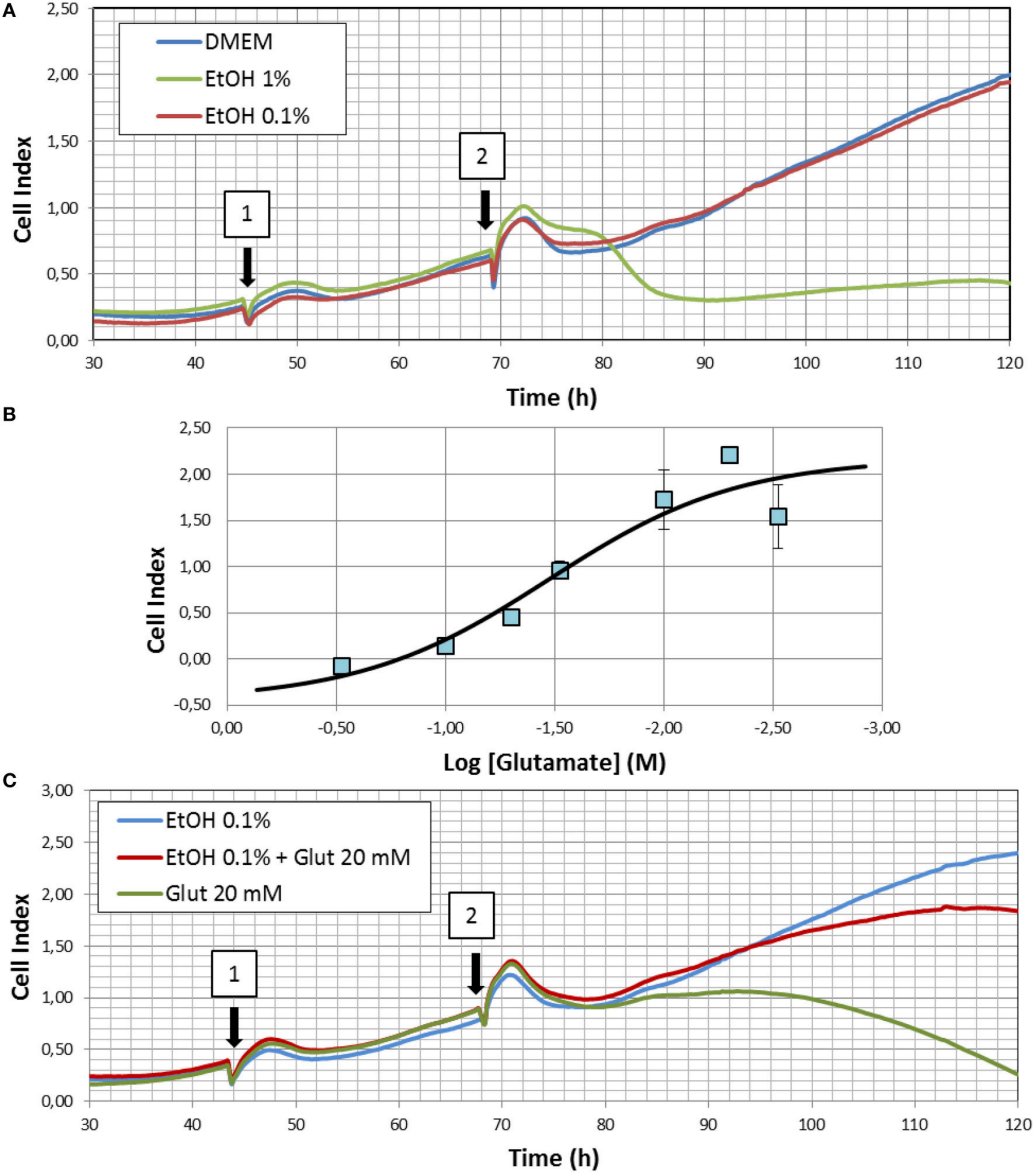
**(A)** Representative experiment showing the effects of 0.1 and 1% ethanol (EtOH) on real time cell proliferation. Arrows indicate the time where media were replaced by either DMEM or EtOH 0.1% in 1 or DMEM, EtOH 0.1, or EtOH 1% in 2. **(B)** Dose-response curve for glutamate toxicity in HT22 cells. Results summarize data from three different experiments and are indicated as mean ± SEM. **(C)** Neuroprotective effects of 0.1% ethanol. Illustrated correspond to a representative experiment of glutamate excitotoxicity in the presence or absence of ethanol. Arrows indicate the time where cells were exposed to either DMEM, EtOH 0.1, or EtOH 0.1% + glutamate in 1, or EtOH 0.1%, EtOH 0.1% + glutamate, or glutamate alone in 2. Three different replicates were performed for experiments illustrated in **(A,C)**.

We finally analyzed the effects of different doses of ethanol treatment on HT22 cells proliferation. Results summarized in Figure 5 shows that HT22 cells reach the maximal cell index (CI_max_) after 120–160 h of seeding in the conditions of the present experiments (Figure 5A). In the presence of 0.1% ethanol, maximal CI values were similar between PBS- and ethanol-treated cells. However, when challenged with a 10-times higher dose of ethanol, a significant reduction of cell proliferation was observed, indicating an important degree of toxicity by this dose of ethanol (Figure 5A), likely caused by an excessive oxidative stress, which overcame the cytoprotective effects of endogenous antioxidant defense induced by low ethanol exposure.

In order to assess whether changes in antioxidant gene expression might confer resistance to toxic oxidative insults, we used glutamate as it has been reported to cause excitotoxicity in HT22 cells (He et al., 2013) and oxidative stress secondary to cysteine depletion (Li et al., 1998). First, using non-linear regression to a four parameter logistic equation, we determined the IC50 value for glutamate toxicity against cell index, and we found a value of 19.5 mM (Figure 5B). Then, we assayed a glutamate concentration of 20 mM in ethanol (0.1%)-treated cells, while monitoring real time cell proliferation. We observed that pre-exposure to 0.1% ethanol significantly prevented glutamate-induced cell death to about 80% of ethanol-treated cells, compared to around 90% cell death in the absence of EtOH. Therefore, we concluded that ethanol efficiently reduced cell death caused by glutamate-induced excitotoxicity.

## Discussion

Our present results demonstrate that treatment of hippocampal HT22 cells with sub-toxic doses of ethanol modifies the expression of specific genes within the classical, thioredoxin, and glutathione systems. We also show that these transcriptional changes are accompanied by consistent modifications in enzyme activities of the three systems and modulation of cellular antioxidant status. First, we observed that ethanol exposure stimulated gene expression of both superoxide dismutase genes (*Sod1* and *Sod2*), and soon increased total SOD activity. The effects of ethanol exposure on the activity of superoxide dismutase are controversial, with reports of an increase (Somani et al., 1996; Enache et al., 2008), no change (Gonenc et al., 2005), or a decrease (Ledig et al., 1981), depending on the brain region, the dose and the duration of ethanol exposure. However, our results are in agreement with previous results showing significant increases in superoxide dismutase and catalase activities in the hippocampus of rats receiving acute intraperitoneal injections of ethanol (1.5 g/kg) (Enache et al., 2008). Similar results were observed in the rat cortex in response to acute ethanol administration (Somani et al., 1996). Interestingly, in our present study, although *Cat* gene expression was not altered by ethanol, a significant increase in enzyme activity was observed by the end of the experiment, which suggests a post-translational modulation of catalase, perhaps associated to increase H_2_O_2_ as a result of SOD activation. The existence of factors modulating catalase activity in response to oxidative stress has been demonstrated in different cell lines (Uenoyama and Ono, 1973; Cao et al., 2003). Several studies have shown that catalase interacts with c-Abl and Arg non-receptor tyrosine kinases, upon activation by H_2_O_2_through a mechanism dependent on protein kinase Cδ (Sun et al., 2000; Cao et al., 2003). The functional significance of these interactions are supported by the demonstration that cells deficient in both c-Abl and Arg exhibit substantial increases in H_2_O_2_ levels and a marked increase in H_2_O_2_-induced apoptosis (Cao et al., 2003).

Within the thioredoxin system we observed that the expression of the gene encoding for cytoplasmic thioredoxin reductase (*Txnrd1*) was significantly increased by ethanol from 6 h, while thioredoxin reductases of mitochondrial and endoplasmic reticulum origins were unaffected or down-regulated. Paralleling these observations, an equivalent increase in tTXNRD activity was observed shortly after the upregulation of *Txnrd1* expression was observed. The finding that tTXNRD activity was augmented is consistent with the fact that cytosolic thioredoxin reductase (encoded by *Txnrd1* gene) is the most abundant isoform in nerve cells (Arnér and Holmgren, 2000; Turanov et al., 2010). Another interesting observation was the dramatic down-regulation of *Txnip* gene expression in response to ethanol. It is known that *Txnip* gene encodes for the thioredoxin interacting protein, a 55 kDa protein that stabilizes reduced thioredoxin and keeps it inactive, thereby functioning as an endogenous inhibitor (Yoshihara et al., 2014). Despite ethanol does not alter thioredoxin genes (*Txn1* and *Txn2*) expression in HT22 cells, reduction in the amount of TXNIP would obviously increase free thioredoxin (Trx) proteins, which would enhance its ROS buffering capacity. This effect is physiologically relevant since mammalian TRXRD reduces oxidized substrates, such as Trx and H_2_O_2_, but also other non-disulfide-containing molecules, such lipid hydroperoxides and other hydroperoxides even independently of Trx, but coupled to selenocysteine or selenodiglutathione reduction (Björnstedt et al., 1995; Arnér and Holmgren, 2000), which notably increases the antioxidant spectrum of TRXR.

A family of proteins related to Trx and regulated by ethanol is peroxiredoxins. Peroxiredoxins contain two conserved cysteines in their active site and utilize Trx as reductant (Rhee et al., 2005), therefore, tightly linked to Trx oxidative status (Hawkes et al., 2013). Currently six peroxiredoxins genes have been described in mammals, with *Prdx2–5* genes being expressed in brain (Hattori et al., 2003; Rhee et al., 2005). We have observed that ethanol treatment brings about a generalized and significant down-regulation (18–39%) of all peroxyredoxin genes. These transcriptional alterations occurred soon after ethanol exposure (from 6 h) and lasted until 48 h. Noticeably, *Srxn1* gene expression (which encodes for sulfiredoxin, the main protein responsible for reactivation of oxidized peroxiredoxins) remained unaffected in response to ethanol. At present, we have no explanation for this concerted reduction of peroxiredoxins but a plausible hypothesis is that by down-regulating their expression (at the same time *Txnrd1* expression increases and *Txnip* expression decreases), reduction of Trx is mostly under the control of thioredoxin reductase activity, with lower amounts of reduced thioredoxin being used to reduce peroxiredoxins. As mentioned before, peroxiredoxins become oxidized upon ROS attack, and their reduction is coupled to oxidation of thioredoxin (Rhee et al., 2005). Holistically, such a mechanism will help to maintain higher cytosolic levels of reduced thioredoxin. Additional experiments, including determination of cytosolic levels of oxidized and reduced thioredoxin in response to ethanol, will help to unravel the physiological significance of these findings.

The last antioxidant system modulated by ethanol in HT22 cells is glutathione/glutaredoxin system. We observed that soon after ethanol exposure, expression levels of *Gpx1* increased, and remained higher than in PBS throughout the experiment. Paralleling these findings, tGPx activity was higher in ethanol-treated cells from 6 h, therefore, correlating changes in expression levels. The increase in tGPx activity was likely attributable to GPx1 activity, since GPx4 activity (and gene expression) remained unaffected. *Gclc* gene expression also exhibited a significant increase in ethanol-treated cells, though in this case the response was delayed compared to *Gpx1* gene, and was observed only after 30 h ethanol treatment. Clearly, this modulation of the glutathione/glutaredoxin system enhances the cellular antioxidant potential attributable to glutathione in neuronal cells, at the same time that provides an efficient strategy to ensure the reduction of oxidized glutathione. Indeed, in the present study, we have observed that levels of reduced (and also oxidized) glutathione remained unchanged in spite of the two ethanol challenges and also that TBARs levels were not different from those observed in PBS-treated cells throughout the experiment. These observations pinpoint to an efficient ROS-buffering capacity in HT22 cells in response to sub-toxic ethanol exposure. Interestingly, in agreement with our results, *in vivo* studies performed in the rat cerebral cortex and corpus striatum have shown that ethanol (1.6 g/kg) significantly increases total GPx (as well as SOD) activity (Somani et al., 1996).

It is well-known that an important effect of ethanol is to increase the generation of ROS, including superoxide and the hydroxyethyl radical (Das and Vasudevan, 2007). Generation of ethanol-derived ROS is expected to interact with cellular targets, particularly with membrane lipids, giving rise to lipid hydroperoxides, which, in turn, initiate a self-propagating oxidative damage (Niki et al., 2005). Therefore, it is expected that levels of lipid-related oxidative species progressively augment as time progresses. Although ROS are generally considered to exert deleterious effects, it is becoming increasingly evident that ROS may serve second messengers implicated in signaling processes, and participate in a number of normal physiological phenomena (Dröge, 2002). Thus, it is likely that ethanol-derived oxidized metabolites may be responsible for triggering transcriptional signals to boost the expression of components of cellular antioxidant systems, as we have previously observed for lipoperoxides derived from docosahexaenoic acid (Casañas-Sánchez et al., 2014). Indeed, It is known that some of the genes studied here, and upregulated by ethanol, contain “antioxidant response elements” (ARE) in their promoter regions (Kaspar et al., 2009; Hawkes et al., 2013). In this sense, recent studies have demonstrated that NF-E2-related factor 2 (Nrf2) is the master transcription factor for the regulation of ARE in different tissues, including the brain (Kobayashi and Yamamoto, 2005; Singh et al., 2010; Zhang et al., 2013).

Overall, we may conclude that sub-toxic ethanol exposure enhances global antioxidant capacity of hippocampal neurons by at least three mechanisms: (1) by enhancing the expression and activity of the generic system through up-regulating superoxide dismutase expression (2) by increasing thioredoxin reductase 1 expression, the most abundant isoform in neuronal tissue, at the same time that down-regulates *Txnip* and peroxiredoxins expression, and (3) by upregulating glutathione peroxidase 1 and glutathione-*S*-reductase genes expression. Taken together these observations led us to envisage that hippocampal cells become more resistant to oxidative insults after being exposed to acute sub-lethal ethanol. Indeed, recent *in vivo* evidence have shown that ethanol pre-conditioning render brain tissue more resistant to oxidative damage in animal models such as the ischemia-reperfusion injury in gerbil and rat models (Liao et al., 2003; Wang et al., 2007), pro-inflammatory lipopolysaccharide-injected rats (Singh et al., 2007), or even mice models of Alzheimers disease (Wang et al., 2006).

Further, in brain cultures, non-neurotoxic alcohol exposure blocks excitotoxic receptor-mediated neurodegeneration triggered by NMDA exposure (Chandler et al., 1993; Cebere and Liljequist, 2003; Belmadani et al., 2004). Effects of alcohol pre-conditioning on inflammatory protein (gp120_*IIIB*_)-induced neurotoxicity have also been explored in organotypic slices of rat hippocampus-entorhinal cortex, two brain regions significantly impacted in Alzheimers disease and other dementias (Collins et al., 2000, 2010). More recently, Muñoz and coworkers have shown that low concentrations of ethanol protect against synaptotoxicity induced by Aβ in hippocampal neurons (Muñoz et al., 2015). In line with this, we show here that ethanol (0.1%) can prevent excitotoxicity induced by glutamate exposure, which is in consonance with the results reported for NMDA exposure in rat primary cultured cells (Chandler et al., 1993) or in rat cerebellar granular cells (Cebere and Liljequist, 2003) in a similar range of concentrations and time-course as used here.

Different mechanisms have been proposed to underlie the neuroprotective effects of sub-lethal ethanol exposure. Overall, an emerging hypothesis is that alcohol pre-conditioning-induced neuronal survival mechanisms involve induction of heat-shock proteins (HSP27 and/or HSP70) upon ROS generation, and that HSP-dependent protection is intimately associated to selective protein kinase C (PKCα and PKCδ) and FAK (focal adhesion kinase) activation, NOS (nitric oxide synthase), the focal adhesion complex, and stabilization of the cytoskeleton (Collins et al., 2009). However, the present study is the first demonstrating that ethanol provides resistance to oxidative insults through mechanisms directly linked to transcriptional modulation of specific components within the set of antioxidant systems. Therefore, under this paradigm, ethanol may be considered an “*Indirect Antioxidant*,” as it has been coined for molecules which although lacking antioxidant activity *per se*, are capable to potentiate cellular antioxidant capacity by enhancing gene expression (Jung and Kwak, 2010). This newly identified neuroprotective (and perhaps anti-excitotoxic) effect of ethanol *in vitro* is clearly hormetic and might be clinically relevant (Rattan, 2004), but certainly it requires further studies before its significance and window of application is completely understood.

## Author contributions

VC and JP performed genetic analyses. DQ and VC performed analyses of enzyme activities. MD designed the study, analyzed the data and drafted the manuscript.

### Conflict of interest statement

The authors declare that the research was conducted in the absence of any commercial or financial relationships that could be construed as a potential conflict of interest.
